# Reductive Heck Reactions of *N*-Methyl-substituted Tricyclic Imides 

**DOI:** 10.3390/molecules15031303

**Published:** 2010-03-04

**Authors:** Gokce Goksu, Nuket Ocal, Dieter E. Kaufmann

**Affiliations:** 1 Department of Chemistry, Faculty of Art and Sciences, Davutpasa Campus, Yildiz Technical University, 34210 Esenler-Istanbul, Turkey; 2 Institute of Organic Chemistry, Leibnizstr. 6, Clausthal University of Technology, D-38678 Clausthal-Zellerfeld, Germany

**Keywords:** homogenous catalysis, palladium, C-C coupling, hydroarylation, heterocycles

## Abstract

The palladium-catalyzed hydroarylation of *N*-methyl-substituted tricyclic imides was studied in order to find a new stereoselective access to a series of new *exo*-aryl(hetaryl)-substituted tricyclic *N*-methylimides.

## 1. Introduction

The imide moiety is an integral structural part of various important bioactive molecules such as fumaramidmycin, granulatimide, isogranulatimide and rebeccamycin. These molecules are reported to exhibit antitumor, anti-inflammatory and antimicrobial activities [1,2,3]. A literature search reveals that certain compounds with antitumor activity, and in particular molecules able to interact with DNA, are characterized by the presence of both an extended π-system and an imide function.

**Figure 1:**
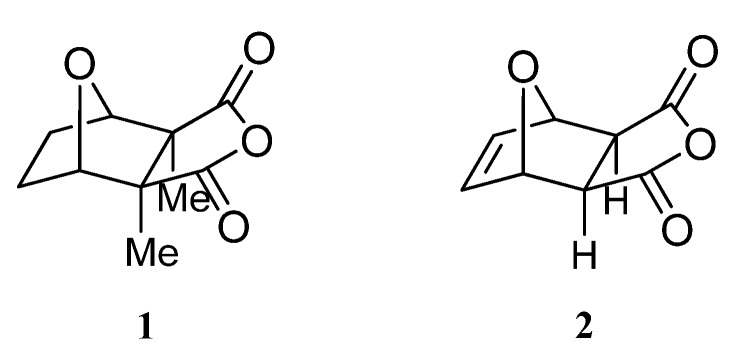
Cantharidin (**1**) and *exo*-5,6-dehydronorcantharidin (**2**).

On the other hand, derivatives of the tricyclic anhydride *exo*-5,6-dehydronorcantharidin (**2**, Figure 1) are also pharmacologically active [4]. Norcantharidin shows a comparable activity with cantharidin (**1**, Figure 1) which is the major effective ingredient in pharmaceuticals for the treatment of certain malignant tumors in China. Compound **2** has been widely employed in clinical practice, as it is less toxic and much easier to synthesize [5,6]. Furthermore, in connection with an additional imide unit this type of structure has recently become an intense research topic in heterocyclic chemistry because of its anti-tumor, anti-virus, analgesic, sedative and fungicidal activities.

Therefore, we became interested in bioactive cantharidin analogues that represent aryl-modified bicyclic imide systems. We first synthesized *endo*-*N*-phenylbicyclo[2.2.1]hept-5-ene-2,3-dicarboximide as the starting material for reductive Heck reactions. In the presence of triphenylarsine as a ligand [7] the palladium-catalyzed hydroarylation of the easily accessible, unsaturated tricyclic *N*-phenyl (or phenyl-substituted) imides has been proven to be a stereoselective, versatile and high-yield approach for the synthesis of the corresponding aryl and heteroaryl derivatives [8,9]. In this paper, we synthesized various *N*-methyl derivatives of the unsaturated imides by a hydroarylation procedure to check the effect on both, the reactivity of the starting materials as well as the bioactivity of the products.

## 2. Results and Discussion

**Scheme 1:**
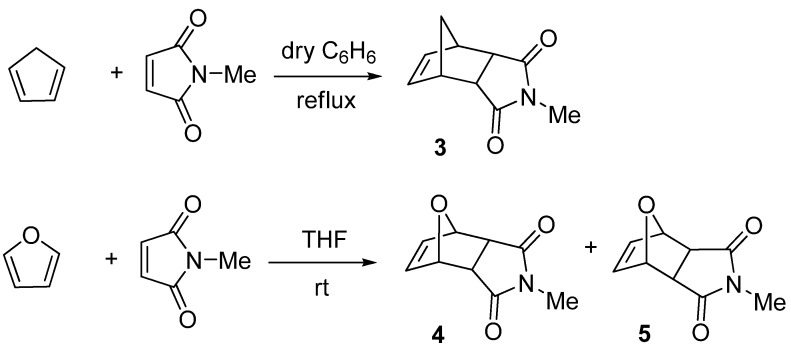
Synthesis of **3–5**.

Our synthesis started with the Diels-Alder reaction of cyclopentadiene and *N*-methylmaleimide in dry benzene under reflux to give *N*-methylbicyclo[2.2.1]hept-5-ene-2-*endo*,3-*endo*-dicarboximide (**3**) as colorless crystals in a yield of 93% [10]. The same reaction was successfully applied to the reaction of furan with *N*-methylmaleimide to give both diastereomers, *N*-methyl-7-oxabicyclo[2.2.1]hept-5-ene-2-*endo*,3-*endo*-dicarboximide (**4**) and *N*-methyl-7-oxabicyclo[2.2.1]hept-5-ene-2-*exo*,3-*exo*-di-carboximide (**5**) in good yieldsafter chromatographic separation [11] (Scheme 1).

**Scheme 2:**
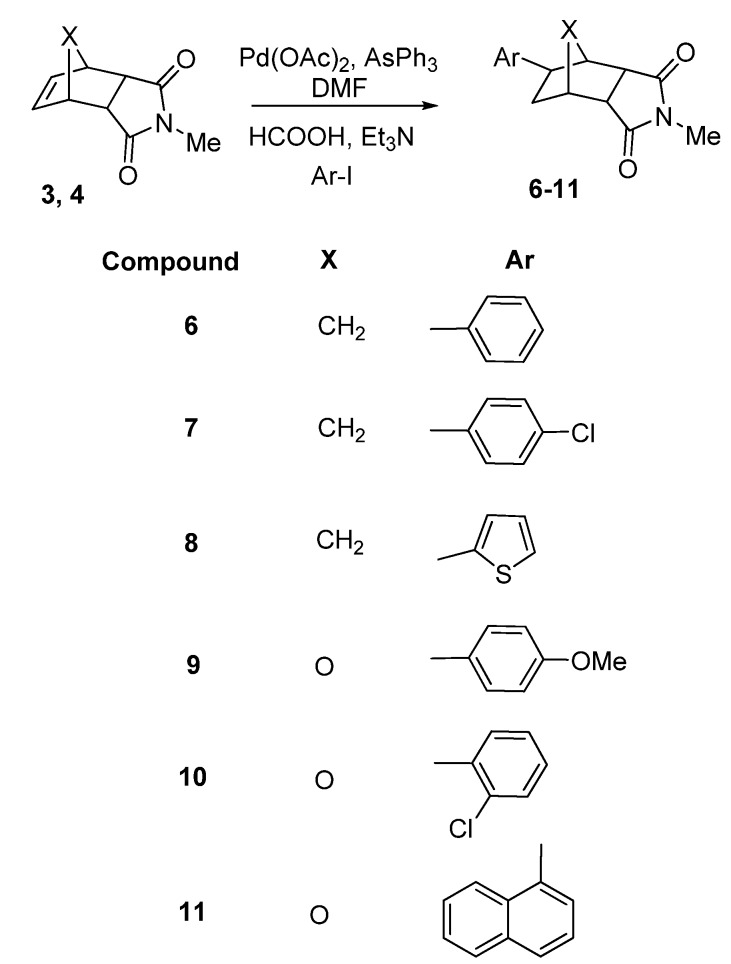
Synthesis of **6–11**.

Treatment of **3** with 1-iodobenzene, 4-chloro-1-iodobenzene and 2-iodothiophene under reductive *Heck* conditions gave the **6, 7** and **8** pure products as *exo*-isomers after chromatographic separation on silica gel in isolated yields of 70–94% (Scheme 2).

**Scheme 3:**
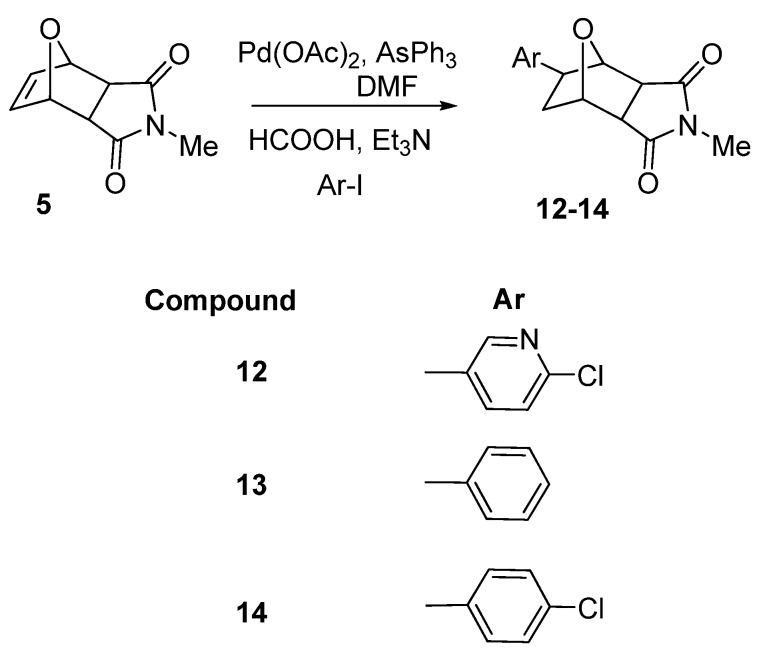
Synthesis of **12**–**14**.

The same reductive Heck arylation conditions were successfully applied to the reaction of **4** with 4-methoxy-1-iodobenzene, 2-chloro-1-iodobenzene and 1-iodonaphthalene to give the new *exo*-arylated heterocycles **9**, **10** and **11** in good yields after chromatographic separation (Scheme 2). We also synthesized **12**, **13** and **14** from **5** with 2-chloro-5-iodopyridine, 1-iodobenzene and 4-chloro-1-iodobenzene prepared asnew 8-*exo*-compounds under the same hydroarylation conditions (Scheme 3).

The new structures were assigned by their ^1^H-NMR data. The stereochemistry was inferred from their NMR spectra including diagnostic spin-spin interactions. The *exo*-position of the C-8 substituent was confirmed by the fact that H_8_ showed no significant interaction with H_1._ The geminal protons at C-9 were identified by vicinal coupling to H_1_. Additionally, H-H COSY spectra showed cross peaks between H_2_ and H_6_ and between H_8_ and H_9_, respectively. In addition to the ^13^C-NMR, DEPT and FTIR spectral data which were in agreement with the proposed structures, the mass spectra of all new compounds showed the expected molecular ion peaks. The data show that independently from the stereochemistry of the starting imide moiety, hydroarylation leads to the formation of the *exo*-arylated products, exclusively.

## 3. Experimental Section

### 3.1. General

All the reactions were carried out under nitrogen atmosphere unless otherwise indicated. Reactions were monitored by thin-layer chromatography (TLC). Visualization of the developed chromatograms were performed either with UV light or KMnO_4_ stain. Products were purified by silica gel chromatography with a solvent gradient of ethyl acetate/*n*-hexane to afford the title compounds. IR spectra were obtained with a Perkin Elmer FT-IR instrument and absorption frequencies are reported in cm^-1^. Melting points were determined with a Gallenkamp digital thermometer. NMR spectra were determined with a Varian-INOVA-500 MHz NMR. 2D NMR experimental studies were measured with a Bruker Ac-400 MHz NMR. TMS (tetramethylsilane) was used as an internal standard and CDCl_3_ was used as the solvent. Signal multiplicities in the NMR spectra are reported as follows: s-singlet, brs-broad singlet, d-doublet, dd-doublet of doublets, m-multiplet. Mass spectra were measured with Agilent 6890N GC-System-5973 IMSD.

### 3.2. General procedure for reductive Heck reactions

A solution of Pd(OAc)_2_ (5.6 mg, 0.025 mmol) and AsPh_3_ (33.7 mg, 0.11 mmol) in anhydrous DMF (3 mL) was stirred under nitrogen at 65 °C for 15 min. Then, alkene (1.0 mmol), Et_3_N (488 μL, 3.5 mmol), aryl(hetaryl) iodide (1.5 mmol) and HCOOH (138 mg, 3 mmol) were added sequentially. The reaction mixture was stirred for 8–24 h. After cooling to room temperature EtOAc and brine were added, the organic layer was separated, dried over MgSO_4_, filtered, and the solvent evaporated. The residue was purified by column chromatography (SiO_2_).


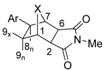


*N-Methyl-exo-8-phenylbicyclo[2.2.1]heptane-3-endo,5-endo-dicarboximide* (**6**). Colorless solid, m.p. 108–110 °C, yield: 70%. IR (ATR) υ: 3029, 2968, 2891, 1763, 1686, 1601, 1491, 1472, 1449, 1427, 1376, 1273, 1258, 1181, 1118, 979, 770, 746 cm^-1^; ^1^H-NMR δ: 1.50 (d, *J* = 10.7 Hz, 1H, H_10a_), 1.68 (ddd, *J* = 15.6, 10.7, 1.9 Hz, 1H, H_10s_), 1.77–1.83 (m, 2H, H_9x_ and H_9n_), 2.68 (dd, *J* = 8.7, 5.8 Hz, 1H, H_8n_), 2.80–2.84 (m, 2H, H_1_ and H_7_), 2.95 (s, 3H, N-CH_3_), 3.08 (ddd, *J* = 14.6, 10.7, 1.9 Hz, 1H, H_2_), 3.14 (dd, *J* = 8.7, 4.8 Hz, 1H, H_6_), 7.08–7.13 (m, 3H, H_ar_), 7.19–7.23 (m, 2H, H_ar_) ppm; ^13^C-NMR δ: 24.65, 32.76, 39.58, 39.82, 42.09, 45.93, 48.76, 49.34, 126.40, 127.26, 128.66, 144.64, 178.34, 178.36 ppm; GC-MS m/z: 255 (M^+^), 143, 113, 66.

*N-Methyl-exo-8-(4-chlorophenyl)bicyclo[2.2.1]heptane-3-endo,5-endo-dicarboximide* (**7**). Colorless solid, m.p. 120–123 °C, yield: 84%. IR (ATR) υ: 2970, 2906, 1768, 1687, 1492, 1454, 1427, 1379, 1275, 1259, 1182, 1138, 1126, 1091, 986, 830, 812, 798 cm^-1^; ^1^H-NMR δ: 1.50 (d, *J* = 9.7 Hz, 1H, H_10a_), 1.65–1.77 (m, 3H, H_10s_, H_9x_ and H_9n_), 2.63–2.67 (m, 1H, H_8n_), 2.77 (d, *J* = 4.8 Hz, 1H, H_1_), 2.82 (brs, 1H, H_7_), 2.95 (s, 3H, N-CH_3_), 3.08 (ddd, *J* = 9.7, 5.8, 2.8 Hz, 1H, H_2_), 3.14 (dd, *J* = 9.7, 5.8 Hz, 1H, H_6_), 7.04 (d, *J* = 8.7 Hz, 2H, H_ar_), 7.17 (d, *J* = 8.7 Hz, 2H, H_ar_) ppm; ^13^C-NMR δ: 24.68, 32.85, 39.52, 39.78, 41.59, 45.88, 48.65, 49.24, 128.60, 128.74, 132.20, 143.12, 178.15, 178.21 ppm; GC-MS m/z: 289 (M^+^), 177, 152, 138, 113, 66.

*N-Methyl-exo-8-(2-thienyl)bicyclo[2.2.1]heptane-3-endo,5-endo-dicarboximide* (**8**). Colorless solid, m.p. 93–96 °C, yield: 94%. IR (ATR) υ: 3068, 2972, 2886, 1762, 1688, 1470, 1430, 1378, 1310, 1272, 1255, 1181, 1119, 1079, 980, 937, 796, 742 cm^-1^; ^1^H-NMR δ: 1.54 (d, *J* = 10.7 Hz, 1H, H_10a_), 1.76 (brd, *J* = 5.8 Hz, 2H, H_9x_ and H_9n_), 1.89 (d, *J* = 10.7 Hz, 1H, H_10s_), 2.81 (brd, *J* = 4.3 Hz, 2H, H_1_ and H_7_), 2.87–2.90 (m, 1H, H_8n_), 2.94 (s, 3H, N-CH_3_), 3.07 (dd, *J* = 8.7, 4.8 Hz, 1H, H_2_), 3.12 (dd, *J* = 8.7, 4.8 Hz, 1H, H_6_), 6.71 (d, *J* = 3.9 Hz, 1H, H_ar_), 6.84 (dd, *J* = 4.8, 3.4 Hz, 1H, H_ar_), 7.06 (dd, *J* = 4.8, 0.9 Hz, 1H, H_ar_) ppm; ^13^C-NMR δ: 24.68, 35.26, 38.27, 39.49, 39.99, 47.26, 48.48, 48.93, 123.56, 123.64, 126.98, 149.61, 178.01, 178.07 ppm; GC-MS m/z: 261 (M^+^), 149, 113, 66.

*N-Methyl-exo-8-(4-methoxyphenyl)-10-oxabicyclo[2.2.1]heptane-3-endo,5-endo-dicarboximide* (**9**). Colorless solid, m.p. 151–153 °C, yield: 91%. IR (ATR) υ: 3041, 2960, 2903, 1766, 1689, 1611, 1512, 1463, 1427, 1374, 1274, 1244, 1180, 1132, 1032, 996, 948, 892, 829 cm^-1^; ^1^H-NMR δ: 1.83 (dt, *J* = 13.6, 4.8 Hz, 1H, H_9x_), 2.03 (dd, *J* = 13.6, 8.7 Hz, 1H, H_9n_), 2.80 (dd, *J* = 8.7, 4.8 Hz, 1H, H_8n_), 2.97 (s, 3H, N-CH_3_), 3.40–3.44 (m, 2H, H_2_ and H_6_), 3.70 (s, 3H, -OCH_3_), 4.65 (brs, 1H, H_7_), 4.96 (brs, 1H, H_1_), 6.74 (d, *J* = 8.7 Hz, 2H, H_ar_), 7.08 (d, *J* = 8.7 Hz, 2H, H_ar_) ppm; ^13^C-NMR δ: 25.07, 37.49, 43.96, 51.59, 51.81, 55.48, 78.11, 84.20, 114.18, 128.48, 136.62, 158.72, 175.63, 175.72 ppm; GC-MS m/z: 287 (M^+^), 259, 147, 68.

*N-Methyl-exo-8-(2-chlorophenyl)-10-oxabicyclo[2.2.1]heptane-3-endo,5-endo-dicarboximide* (**10**). Colorless solid, m.p. 122–123 °C, yield: 65%. IR (ATR) υ: 3019, 2973, 2948, 1775, 1693, 1471, 1432, 1379, 1311, 1274, 1129, 994, 954, 835 cm^-1^; ^1^H-NMR δ: 1.76–1.80 (m, 1H, H_9x_), 2.12 (dd, *J* = 13.6, 8.7 Hz, 1H, H_9n_), 2.99 (s, 3H, N-CH_3_), 3.42 (dd, *J* = 8.7, 4.8 Hz, 1H, H_8n_), 3.45-3.47 (m, 2H, H_2_ and H_6_), 4.80 (brd, *J* = 4.8 Hz, 1H, H_1_), 4.98 (brs, 1H, H_7_), 7.08–7.16 (m, 2H, H_ar_), 7.26 (d, *J* = 8.7 Hz, 1H, H_ar_), 7.32 (d, *J* = 8.7 Hz, 1H, H_ar_) ppm; ^13^C-NMR δ: 23.85, 35.24, 39.43, 50.38, 50.54, 77.10, 81.65, 126.28, 126.44, 126.92, 128.32, 132.45, 140.07, 173.90, 174.37 ppm; GC-MS m/z: 291 (M^+^), 256, 228, 151, 112, 68.

*N-Methyl-exo-8-(1-naphthyl)-10-oxabicyclo[2.2.1]heptane-3-endo,5-endo-dicarboximide* (**11**). Colorless solid, m.p. 170–172 °C, yield: 62%. IR (ATR) υ: 2987, 2964, 1770, 1690, 1597, 1508, 1428, 1375, 1314, 1271, 1136, 1065, 1001, 954, 894, 802, 786 cm^-1^; ^1^H-NMR δ: 2.06–2.16 (m, 2H, H_9x_ and H_9n_), 3.05 (s, 3H, N-CH_3_), 3.48–3.56 (m, 2H, H_2_ and H_6_), 3.72 (dd, *J* = 8.7, 4.8 Hz, H_8n_), 4.83 (d, *J* = 5.8 Hz, 1H, H_7_), 5.04 (t, *J* = 5.8 Hz, 1H, H_1_), 7.36–7.38 (m, 2H, H_ar_), 7.40–7.44 (m, 1H, H_ar_), 7.48–7.51 (m, 1H, H_ar_), 7.66 (d, *J* = 6.1, 2.9 Hz, 1H, H_ar_), 7.78 (d, *J* = 8.7 Hz, 1H, H_ar_), 7.86 (d, *J* = 8.7 Hz, 1H, H_ar_) ppm; ^13^C-NMR δ: 25.20, 35.22, 39,75, 51.82, 51.83, 78.36, 83.26, 122.92, 123.39, 125.66, 125.93, 126.77, 127.71, 129.23, 131.57, 134.10, 138.74, 175.73, 176.04 ppm; GC-MS m/z: 307 (M^+^), 167, 152, 112, 68.

*N-Methyl-exo-8-(6-chloro-3-pyridinyl)-10-oxabicyclo[2.2.1]heptane-3-exo,5-exo-dicarboximide* (**12**). Colorless solid, m.p. 202–204 °C, yield: 41%. IR (ATR) υ: 3060, 2993, 1770, 1688, 1566, 1454, 1433, 1381, 1282, 1266, 1130, 1028, 1003, 887, 828, 784 cm^-1^; ^1^H-NMR δ: 1.80 (dt, *J* = 13.1, 4.8 Hz, 1H, H_9x_), 2.20 (dd, *J* = 13.1, 8.7 Hz, 1H, H_9n_), 2.91 (s, 3H, N-CH_3_), 2.95 (d, *J* = 6.8 Hz, 1H, H_2_), 2.98 (d, *J* = 4.8 Hz, 1H, H_8n_), 3.00 (d, *J* = 6.8 Hz, 1H, H_6_), 4.68 (s, 1H, H_7_), 4.98 (d, *J* = 5.3 Hz, 1H, H_1_), 7.22 (s, 1H, H_ar_), 7.53 (dd, *J* = 8.7, 2.4 Hz, 1H, H_ar_), 8.18 (d, *J* = 2.4 Hz, 1H, H_ar_) ppm; ^13^C-NMR δ: 25.38, 40.29, 44.35, 49.86, 50.12, 79.21, 84.51, 124.72, 137.51, 138.74, 148.74, 150.42, 176.43, 176.74 ppm; GC-MS m/z: 292 (M^+^), 180, 153, 117, 68.

*N-Methyl-exo-8-phenyl-10-oxabicyclo[2.2.1]heptane-3-exo,5-exo-dicarboximide* (**13**). Colorless solid, m.p. 137–138 °C, yield: 79%. IR (ATR) υ: 3062, 2956, 1768, 1683, 1479, 1431, 1386, 1288, 1136, 1008, 986, 888, 837, 763, 745 cm^-1^; ^1^H-NMR δ: 1.89 (dt, *J* = 12.6, 4.8 Hz, 1H, H_9x_), 2.15 (dd, *J* = 12.6, 8.7 Hz, 1H, H_9n_), 2.90 (s, 3H, N-CH_3_), 2.91-2.98 (m, 3H, H_8n_, H_2_ and H_6_), 4.72 (s, 1H, H_7_), 4.93 (d, *J* = 4.8 Hz, 1H, H_1_), 7.13–7.17 (m, 3H, H_ar_), 7.19–7.23 (m, 2H, H_ar_) ppm; ^13^C-NMR δ: 25.29, 40.29, 47.59, 50.01, 50.43, 79.28, 84.92, 127.06, 127.33, 128.90, 144.30, 176.94, 177.25 ppm; GC-MS m/z: 257 (M^+^), 157, 117, 68.

*N-Methyl-exo-8-(4-chlorophenyl)-10-oxabicyclo[2.2.1]heptane-3-exo,5-exo-dicarboximide* (**14**). Colorless solid, m.p. 174–175 °C, yield: 82%. IR (ATR) υ: 2991, 1770, 1692, 1491, 1432, 1376, 1283, 1183, 1131, 1089, 1000, 979, 883, 823, 808, 779 cm^-1^; ^1^H-NMR δ: 1.82 (dt, *J* = 12.6, 4.8 Hz, 1H, H_9x_), 2.16 (dd, *J* = 12.6, 8.7 Hz, 1H, H_9n_), 2.90 (s, 3H, N-CH_3_), 2.92 (d, *J* = 6.8 Hz, 1H, H_2_), 2.94 (d, *J* = 4.8 Hz, 1H, H_8n_), 2.96 (d, *J* = 6.8 Hz, 1H, H_6_), 4.68 (s, 1H, H_7_), 4.94 (d, *J* = 4.8 Hz, 1H, H_1_), 7.10 (d, *J* = 8.7 Hz, 2H, H_ar_), 7.18 (d, *J* = 8.7 Hz, 2H, H_ar_) ppm; ^13^C-NMR δ: 25.32, 40.35, 46.99, 49.95, 50.30, 79.22, 84.80, 128.70, 129.01, 132.91, 142.80, 176.78, 177.06 ppm; GC-MS m/z: 291 (M^+^), 191, 151, 112, 68.

## 4. Conclusions

In summary, in the presence of triphenylarsine as a ligand the palladium-catalyzed hydroarylation of the readily accessible *N*-substituted tricyclic imides **3, 4** and **5** was shown to be a stereoselective, versatile and high yield approach to the synthesis of aryl and heteroaryl derivatives of tricyclic imides **6–14**. The above approach has been proved very useful for the construction of novel heterocycles of potential pharmacological interest.

## References

[1] Brana M.F., Gradillas A., Gomez A., Acero N., Llinares F., Munoz-Mingarrro D., Abradelo C., Rey-Stolle F., Yuste M., Campos J., Gallo M.A., Espinosa A. (2004). Synthesis, biological activity, and quantitative structure−activity relationship study of azanaphthalimide and arylnaphthalimide derivatives. J. Med. Chem..

[2] Sondhi S.M., Rani R., Roy P., Agrawal S.K., Saxena A.K. (2009). Microwave-assisted synthesis of *N*-substituted cyclic imides and their evaluation for anticancer and anti-inflammatory activities. Bioorg. Med. Chem. Lett..

[3] Kossakowski J., Jarocka M. (2001). Synthesis of new N-substituted cyclic imides with an expected anxiolytic activity. XVII. Derivatives of 1-ethoxybicyclo[2.2.2]oct-5-one-2,3-dicarboximide. II Farmaco.

[4] Hart M.E., Chamberlin A.R., Walkom C., Sakoff J.A., McCluskey A. (2004). Modified norcantharidins: synthesis, protein phosphatases 1 and 2A inhibition, and anticancer activity. Bioorg. Med. Chem. Lett..

[5] Deng L., Yongzhou H. (2007). 1,3-Dipolar cycloaddition reaction: synthesis and configuration of norcantharidin derivatives of substituted aromatic amines. J. Heterocycl. Chem..

[6] Deng L.-P., Liu F.-M., Wang H.-Y. (2005). 1,3-Dipolar cycloaddition reaction: synthesis of novel 5,6-dehydronorcantharidin derivatives of substituted aromatic amines with potential antitumor activities. J. Heterocycl. Chem..

[7] Namyslo J.C., Kaufmann D.E. (1999). Triphenylarsine as an efficient ligand in the Pd-catalyzed synthesis of Epibatidine and analogs. Synlett.

[8] Goksu G., Gul M., Ocal N., Kaufmann D.E. (2008). Hydroarylation of bicyclic, unsaturated dicarboximides: access to aryl-substituted, bridged perhydroisoindoles. Tetrahedron Lett..

[9] Celik C., Kulu I., Ocal N., Kaufmann D.E. (2009). Domino-Heck reactions of carba- and oxabicyclic, unsaturated dicarboximides: Synthesis of aryl-substituted, bridged perhydroisoindole derivatives. Helv. Chim. Acta.

[10] Birney D., Lim T.-K., Koh Joanne H.P., Pool B.R., White J.M. (2002). Structural investigations into the retro-Diels-Alder reaction. Experimental and theoretical studies. J. Am. Chem. Soc..

[11] By G., Yit W., Pool B.R., White J.M. (2008). Structural studies on cycloadducts of furan, 2-methoxyfuran, and 5-trimethylsilylcyclopentadiene with maleic anhydride and *N*-methylmaleimide. J. Org. Chem..

